# Emerging Two-Dimensional Materials-Based Electrochemical Sensors for Human Health and Environment Applications

**DOI:** 10.3390/nano13040780

**Published:** 2023-02-20

**Authors:** Muhammad Atif Khan, Faizan Ramzan, Muhammad Ali, Muhammad Zubair, Muhammad Qasim Mehmood, Yehia Massoud

**Affiliations:** Innovative Technologies Laboratories (ITL), King Abdullah University of Science and Technology (KAUST), Thuwal 23955, Saudi Arabia

**Keywords:** 2D materials, glucose detection, pesticides, nitrate and nitrite detection, electrochemical sensors

## Abstract

Two-dimensional materials (2DMs) have been vastly studied for various electrochemical sensors. Among these, the sensors that are directly related to human life and health are extremely important. Owing to their exclusive properties, 2DMs are vastly studied for electrochemical sensing. Here we have provided a selective overview of 2DMs-based electrochemical sensors that directly affect human life and health. We have explored graphene and its derivatives, transition metal dichalcogenide and MXenes-based electrochemical sensors for applications such as glucose detection in human blood, detection of nitrates and nitrites, and sensing of pesticides. We believe that the areas discussed here are extremely important and we have summarized the prominent reports on these significant areas together. We believe that our work will be able to provide guidelines for the evolution of electrochemical sensors in the future.

## 1. Introduction

Since a single layer of graphene was first isolated from bulk graphite in early 2004, there has been a significant amount of interest from the scientific community in graphene and other 2DMs [1,2,3,4,5]. Initially, the major focus was on the applications of 2DMs in solid-state electronic devices to help extend Moore’s law by overcoming the limitations of silicon technologies [6]. However, because of their unique electronic, physical, chemical and thermal properties, researchers from different fields have started to explore 2DMs for various applications such as sensors [7,8], printed electronics [9,10], photonics [11,12,13], batteries [14,15], and biomedicine [16,17]. Among sensors, electrochemical sensors are particularly important. The electrochemical sensors deal with various areas that are directly related to human life and human health [18,19,20]. The unique properties of 2DMs make them suitable for electrochemical sensing and enhancing the performance of existing electrochemical sensors [21,22,23]. In principle, it is anticipated that a decrease in the level of dimensionality of such sensing material will result in an improvement in the sensor’s response. Among reduced-dimensioned materials, 2DMs are preferred over 0D and 1D materials because they are compatible with thin-film silicon growth and fabrication technologies [24,25]. Further, their properties can be tuned by techniques such as defect engineering, formation of the van der Waals heterostructure, and doping [26,27,28]. 2DMs offer several other advantages to become the material of choice for future electrochemical sensors. Their surface-to-volume ratio (SVR) is very high, especially for monolayer materials, thus enhancing their chemical reactivity and ensuring maximum interaction between the stimulus and 2D sensing material [29]. They offer a tunable conductivity, which is critical for electrochemical sensors. The conductivity can be modulated by electrochemical interaction, the number of layers, doping, or electrostatic gating [30,31,32]. Moreover, the band gap is also variable and changes with the number of layers as well as external influences such as mechanical stress, etc. [33,34,35]. They also offer mechanical strength and flexibility, which are essential for electrochemical sensors [36]. Owing to these unique advantages, 2DMs can become the main sensing material in various future electrochemical sensors. The family of 2DMs comprises different types of materials such as insulators [37,38,39,40], semiconductors [41,42,43,44,45], semimetals [46,47,48], conductors [49,50,51], and superconductors [52,53,54]. Among them graphene, transition metal dichalcogenides, and MXenes are vastly studied [55,56,57]. In 2DMs, graphene has become a “wonder substance” because of the extraordinary chemical and physical properties that it possesses. At room temperature (RT), in which the hole and electron mobilities are roughly equivalent, the mobility of graphene was 15,000 cm${}^{2}$ V${}^{-1}$ s${}^{-1}$. Graphene is the most attractive 2DM since it has a straight bandgap and a zero bandgap. Several review articles on 2D materials-based electrochemical sensors have been reported in the literature. For example, Reddy et al. reviewed the reports on graphene and its derivatives for electrochemical biosensors [58]. Li et al. focused on graphene-based sensors for nitrite and nitrate detection only [59]. Rhouati et al. reviewed the MXenes-based sensors for the detection of environmental pollutants such as pesticides and heavy metal ions [60]. Amali et al. discussed various nanomaterials for nitrate detection in their review [61]. Carbone et al. summarized graphene-based sensors for the detection of glucose [62]. However, there is still room for a comprehensive study that discusses multiple 2D materials-based sensors that affect several aspects of human life. Here, we have provided a selective overview of the important 2DMs-based electrochemical sensors that are closely related to human life and human health. We have explored areas such as glucose detection in human blood, detection of nitrates and nitrites, and sensing of pesticides.

## 2. 2DMs in Electrochemical Sensing for Glucose Level in Blood

Diabetes is a common cause of early death worldwide. Continuous monitoring of glucose levels is essential for its cure. Accurate glucose level monitoring has remained a hot topic in research and numerous methods have been reported for invasive as well as non-invasive monitoring of glucose levels. Electrochemical sensors for glucose detection can be characterized into two types: enzyme-based and non-enzymatic. For such sensors, 2DMs possess the advantage of a high SVR, thus allowing maximum surface reactions and charge transfer. Therefore, several 2DMs such as graphene, GO, rGO, TMDs, and MXene and their composites are vastly studied in electrochemical sensors for glucose detection.

**Figure 1:**
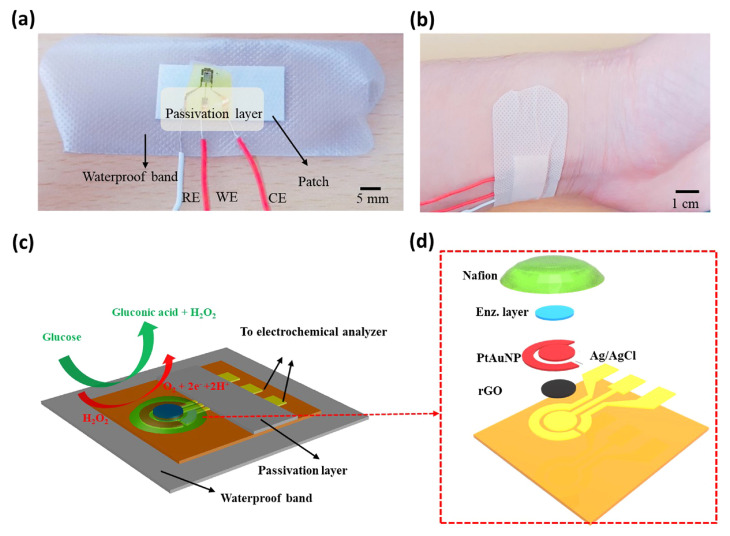
Photos and schematic of the glucose sensor (**a**) Picture of the sensor; (**b**) Sensor mounted on the body; (**c**) Schematic explaining the sensing mechanism; (**d**) Exploded view. Reprinted with permission from Ref. [64].

Razmi et al. used graphene quantum dots (GQD) as a substrate for the enzymatic glucose sensors. The sensor exhibits a sensitivity (ST) of 85 µA mM${}^{-1}$ cm${}^{-2}$ over a detection range (DR) of 5–1270 µM. For better readability, we have abbreviated µA mM${}^{-1}$ cm${}^{-2}$ as AMc throughout the paper. The GQDs possess high porosity and biocompatibility, resulting in better enzyme absorption [63]. Xuan et al. demonstrated a non-invasive wearable sensor based on rGO composite to detect glucose levels using human sweat, as shown in Figure 1. This sensor has a DR of 0–2.4 mM with a ST of 48 AMc. The sensor benefits from a large surface area (SA) and good electroactivity of rGO [64].

**Figure 2:**
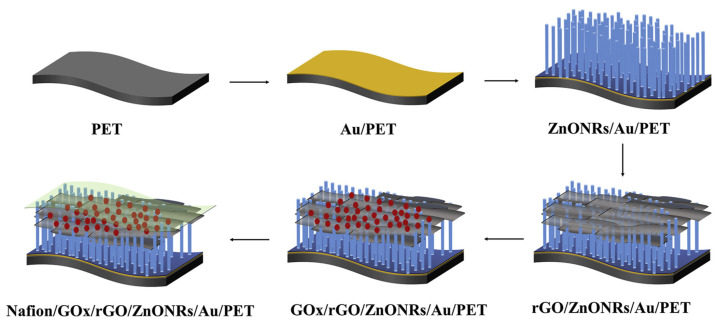
Schematic illustrating the steps of fabrication. Reprinted with permission from Ref. [66].

Hossain et al. reported Pt NPs decorated graphene and carbon nanotubes electrode for glucose sensing. Glucose oxidase was stationed on the electrode surface for the detection of glucose level and a ST of 26.5 AMc was obtained in a DR of 0.5 mM to 13.5 mM. The biosensors were immune to interference species and exhibited stable results when temperature and pH were varied [65]. Mao et al. fabricated a flexible glucose sensor on polyethylene terephthalate (PET) substrate based on ZnO nanorods and rGO, as shown in Figure 2. The sensor benefited from the high electron transfer capability of rGO and produced a low resistance of 0.1234 kΩ and a ST of 5.40 AMc. The sensor works efficiently until 10 bending cycles; however, its performance starts to deteriorate after that [66].

Glucose sensors based on TMDs are also present in the literature. Most of the TMDs capture the target molecules by physisorption caused by the van Der Waals force. TMDs are often decorated with metal NPs to further enhance the interaction between the target species and TMDs. For example, Huang et al. studied Cu NPs decorated MoS${}_{2}$. The sensor was characterized by cyclic voltammetry and amperometric measurements. The sensor exhibits a ST of 1055 AMc with a DR of up to 4 mM [67]. Su et al. detected the glucose on a glassy carbon (GC) electrode with Au NPs decorated MoS${}_{2}$ nanosheets. The use of MoS${}_{2}$ enabled the direct detection of glucose levels, eliminating the need for an electron mediator. The sensors can give stable and reproducible results for a DR of 10 to 300 µM [68]. Similarly, Parlak et al. reported sensors based on Au NPs decorated MoS${}_{2}$ sheets. The Au NPs improve the electrochemical properties such as current density and mobility, thus resulting in a ST of 13,800 AMc and linear response for the DR of 0.25 to 13.2 mM [69].

**Figure 3:**
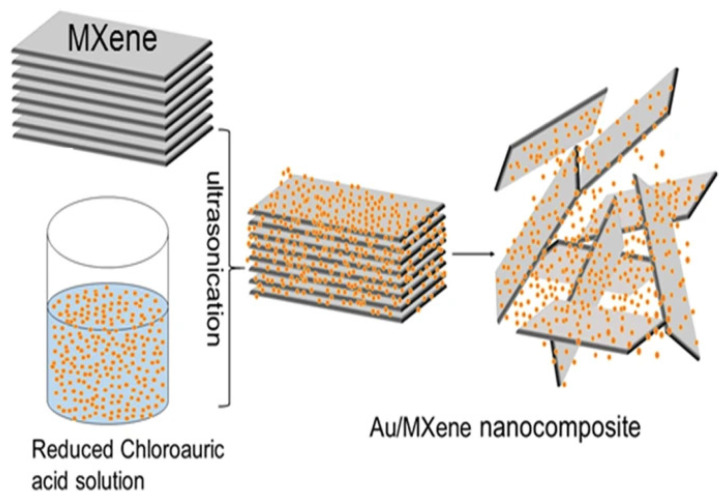
Preparation method for nanocomposite of gold nanoparticles and MXene [70].

Likewise, MXenes also have a strong potential for electrochemical sensing owing to their surface bonding that terminates in electronegative species such as O, OH, and F. These hydrophilic species can adsorb the target molecules for efficient sensing and detection. Moreover, their performance can be further improved by making hybrid combinations with materials such as metal NPs, enzymes, and metal oxides, etc. Rakhi et al. utilized a composite of Ti${}_{3}$C${}_{2}$Tx (MXene) and Au NPs for their amperometric glucose sensor. The Au NPs facilitated the charge transfer between GOx and the electrode. The nanocomposite preparation process has been illustrated in Figure 3. The sensor was prepared on a GC electrode and exhibited a ST of 4.2 AMc for the DR of 0.1 to 18 mM [70].

**Figure 4:**
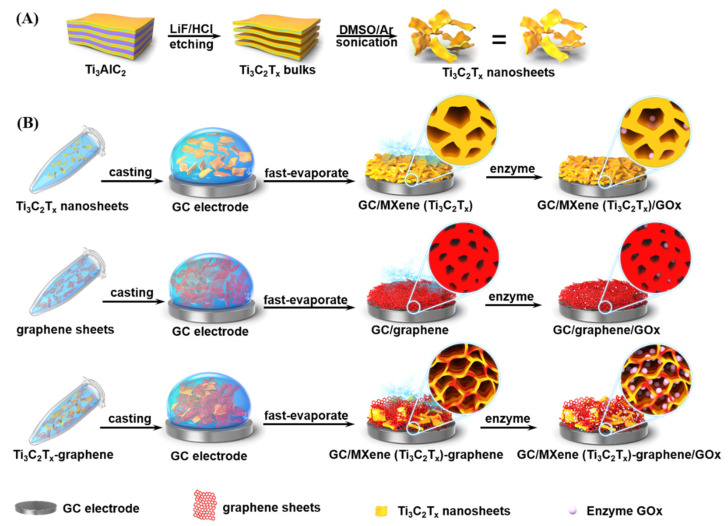
Schematic illustrating the synthesis of (**A**) Ti${}_{3}$C${}_{2}$Tx nanosheets; (**B**) Ti${}_{3}$C${}_{2}$Tx Film, Graphene Film, and hybrid of MXene and graphene. Reprinted with permission from Ref. [71].

To improve the capturing and immobilization of GOx, Gu et al. produced a 3D porous film based on graphene and MXene (Ti${}_{3}$C${}_{2}$Tx). This hybrid film offers a porous and hydrophilic environment that can be further tuned by the ratio of graphene and MXene, as shown in Figure 4. The obtained ST was 20.16 AMc [71].

**Figure 5:**
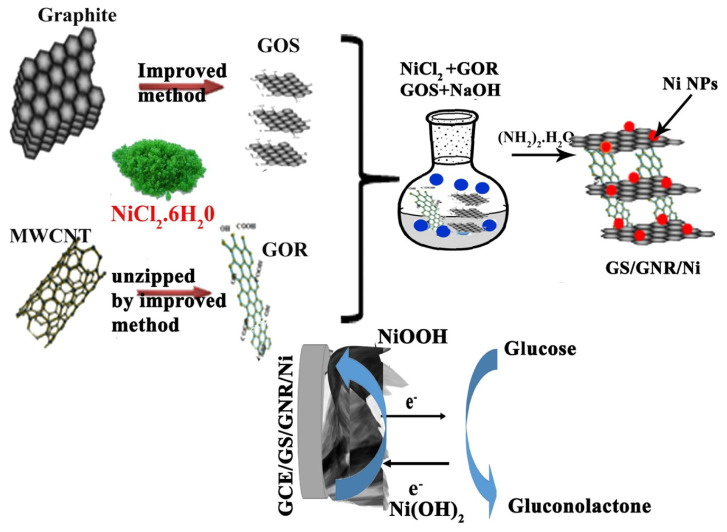
Schematic synthesis and sensing mechanism. Reprinted with permission from Ref. [72].

Until now we have focused on enzymatic glucose sensors based on 2DMs. From here onward we shall discuss the non-enzymatic sensors. Although most glucose sensors are enzyme based, there are several drawbacks associated with such sensors. This includes the instability of enzymes under extreme environments, pH and temperature-dependent output, and sophisticated conditions required for the storage and operation of enzymes. Non-enzymatic glucose sensors can overcome the intrinsic drawbacks present in enzymatic sensors. Glucose sensors that are non-enzymatic depend on the immediate electrochemical oxidation of the blood glucose to function. Jothi et al. prepared an electrode from a combination of nickel NPs, graphene sheet, and nanoribbon electrodes for their sensor, as shown in Figure 5. This hybrid material has a porous structure with a large SA for an enhanced electrocatalytic response of glucose. A linear amperometric response was obtained for the DR of 5 nM–5 mM and a ST of 2300 AMc [72].

**Figure 6:**
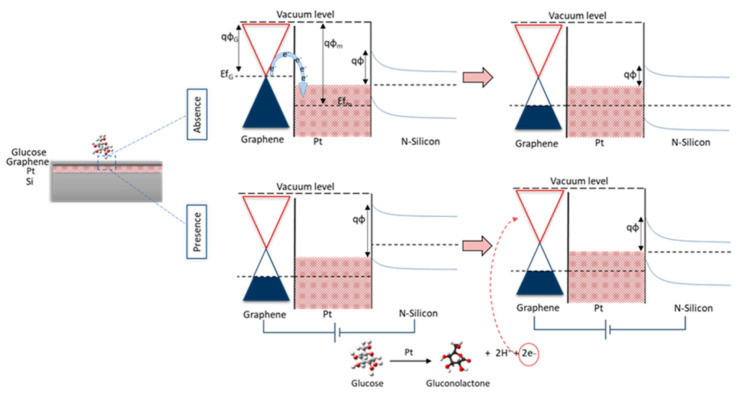
The working of the device explained by the band diagram [73].

Sakr et al. studied a non-enzymatic sensor based on graphene-platinum oxide-Silicon heterostructure. The main sensing mechanism was the variation caused by the glucose in the Schottky barrier, which was present at the graphene-Pt interface, as shown in Figure 6. The obtained results can be tuned by changing platinum oxide and graphene thickness. The maximum obtained ST is 30 AMc [73].

Ayranci et al. demonstrated that monodisperse Pt-Ni composites decorated on rGO produce smaller nanocomposites for glucose detection. A ST of 171.92 µA mM${}^{-1}$ cm${}^{-2}$ for a linear DR of 0.02–5.0 mM was obtained. The bimetallic Pt-Ni exhibited higher electronic and catalytic properties and gave consistent repeatable results [74]. Deshmukh et al. enhanced the electrochemical activity of polyaniline and polyaniline/rGO composite for glucose detection by decorating them with Ag NPs. This enhancement has been attributed to the interaction between nitrogen atoms of polyaniline and Au NPs that increase the exchange rate of electrons, and the synthesis of polyaniline/rGO composite along with Ag NPs decoration. The obtained ST was 2766.4 AMc for a DR of 50–0.1 µM with a DL of 0.79 mM [75]. In TMDs, multiple studies have researched MoS${}_{2}$-based glucose sensors. The research was mainly focused on enhancing the electrical conductivity of MoS${}_{2}$ as well as increasing the exposed catalytic sites. Geng et al. studied Ni-doped MoS${}_{2}$/rGO composites for sensing glucose levels. These composites possess a large SA along with a lot of exposed catalytic sites, and suitable electrical conductivity, which results in the good electrocatalytic oxidation of glucose. A ST of 256.6 AMc for the DR of 0.005–8.2 mM with a DL of 2.7 µM was achieved [76]. Wu et al. performed electrochemical reduction of MoS${}_{2}$ in sodium chloride solution to reduction-MoS${}_{2}$ and used it for glucose detection. After reducing MoS${}_{2}$, improvements in overall conductivity, electron transfer, and electrochemical activity were observed [77].

**Figure 7:**
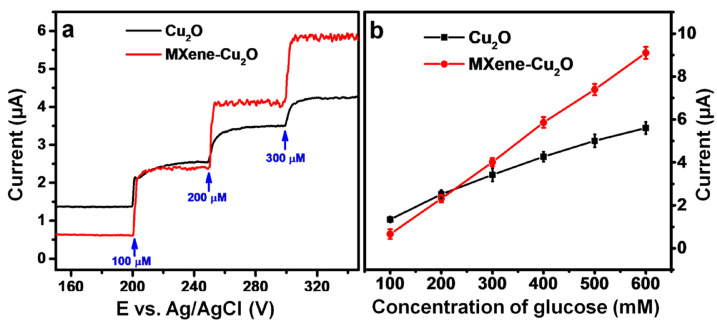
(**a**) Chronoamperometry response and (**b**) Glucose concentration versus current for Cu${}_{2}$O and MXene-Cu${}_{2}$O composite. Reprinted with permission from Ref. [82].

Sensors based on other TMDs such as Ni${}_{3}$Te${}_{2}$, NiSe${}_{2}$, MoSe${}_{2}$, and CoTe${}_{2}$ are also reported [78,79,80,81]. Gopal et al. prepared a Ti${}_{3}$C${}_{2}$Tx-Cu${}_{2}$O (MXene-Cu${}_{2}$O) composite using the wet precipitation method for glucose sensing. The composite exhibited high selectivity and high electrocatalytic activity as compared to bare MXene or Cu2O, as shown in Figure 7. The reported ST was 11.061 AMc for a DR of 0.01–30 mM with a DL of 2.83 µM [82]. Li et al. reported Nickel–Cobalt layered double hydroxide and MXene (Ti${}_{3}$C${}_{2}$Tx) composites for glucose detection. A ST of 64.75 AMc with a DL of 0.53 µM has been reported. The MXene-based composite offers several advantages such as large SA, facile diffusion, and electron transfer [83]. Li et al. used Cu-Cu${}_{2}$O NPs on Ti${}_{3}$C${}_{2}$Tx MXene and used it for H${}_{2}$O${}_{2}$ detection. This study can be extended for an MXene-based electrochemical sensor for glucose detection [84].

The above studies clearly manifest the potential of 2DMs to be used in commercial glucose sensors. The discussion about glucose sensors based on 2DMs has been summarized in Table 1.

## 3. 2DMs Based Composites for Electrochemical Detection of Nitrates and Nitrites

Nitrates are a vital part of the ecosystem and are naturally produced by the nitrogen cycle [85]. Nitrates in the soil as well as water are essential for the growth of plants and aquatic vegetation [86]. Nitrates are vital in regulating different functions of the human body, and drinking water and leafy vegetables are key sources of nitrates in the human diet. However, the excess of nitrates in soil and water can have adverse impacts on human body [87]. The major sources of the overabundance of nitrates in the ecosystem are fertilizers, meat preservatives, and the chemical industry [88]. Due to these adverse effects of nitrates on humans as well as other forms of life, it becomes essential to develop electrochemical sensors for the accurate monitoring of the nitrate level. These sensors can be enzymatic or non-enzymatic. Enzymatic sensors rely on Nitrate reductase (NiR), an enzyme that facilitates the catalytic conversion of nitrate to nitrite [89]. The immobilization of NiR enzyme on the electrode surface of the electrochemical sensor is the key requirement for such an enzymatic sensor. The large SA and high surface energy of 2DMs help in fulfilling this requirement, thus making 2DMs important materials for future practical sensors. Ali et al. reported GO-based nitrate sensors on the surface of a bioelectrode [90] The bioelectrode comprises GO sheets coated on a gold layer on a stretched PDMS. The oxygenated functional groups of GO coupled with the ridges that form by pre-stretching resulted in a better immobilization of NiR enzyme and reduction of nitrate. A ST of 0.224 µA Lmol${}^{-1}$ cm${}^{-2}$ was obtained at a pre-stretching of 8%. Pre-stretching enhanced the ST by five times. Ali et al. utilized TiO${}_{2}$ nanofibers modified graphene foam for the detection of nitrate [91]. The porous structure of foam along with the electron transfer ability of TiO${}_{2}$ resulted in enhanced performance of the sensor. A ST of 0.316 kΩ/µM/cm${}^{2}$ with a fast detection time of 87 s was achieved. Bagheri et al. reported Copper nanoparticles/CNTs/rGO nanocomposite on the GC electrode for nitrate sensing [92]. The nanocomposite enhanced the performance of bare GC electrode with a DL of 20 nM.

**Figure 8:**
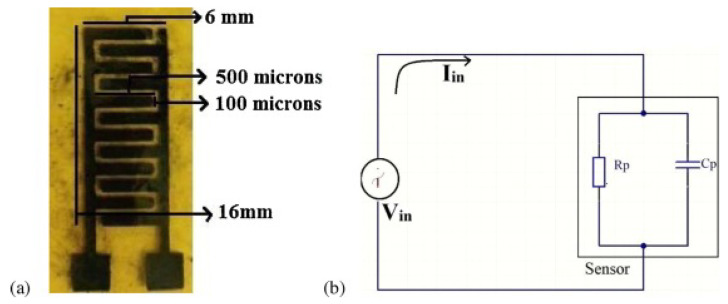
(**a**) Picture of the fabricated device. (**b**). The equivalent circuit of the device. Reprinted with permission from Ref. [94].

Among non-enzymatic sensors, Oznülüer et al. demonstrated the operation of graphene-modified copper electrodes for the detection of nitrate [93]. The graphene modification improved the surface properties of copper electrodes and resulted in a ST of 173. AMc with a DL of 10.0 µM. Alahi et al. demonstrated a graphene-based interdigitated sensor for nitrate sensing [94]. The reported sensor along with the equivalent circuit is shown in Figure 8. The sensor was based on IoT and had connectivity to the Wi-Fi as well. The sensor could be used to detect nitrate in water and had a DL of 1 mg/L.

Wang et al. studied an array of graphene electrodes for selectively detecting nitrate [95]. To improve the selectivity, an ionophore film was coated on top of graphene. The sensor exhibits good selectivity in the DR of 10–1000 ppm. Garland et al. utilized graphene on a flexible polyimide substrate for nitrate detection [96]. The fabrication was carried out using the laser writing method. A ST of − 54.8 ± 2.5 mV/dec with a DL of 20.6 ± 14.8 µM was achieved. The major advantage of this method is one-step fabrication, eliminating the need of metal NPs as well.

Among other 2Dms, Ali et al. reported a composite formed from poly(3-octyl-thiophene) and MoS${}_{2}$ for nitrate detection in the soil [97]. The composite has good ST and selectivity due to its hydrophobicity and redox properties. This sensor gives stable results for up to four weeks when deployed in the soil for nitrate sensing. The sensor exhibits a ST of 64 mV/decade with a DR of 1–1500 ppm and a DL of 1.3 ppm. Similar to nitrate, nitrite also has adverse effects on human health. Nitrite can produce cancer-causing N-nitrosamine in the body. It can also disturb the oxygen-carrying operation of hemoglobin in the blood. The maximum allowable amount of nitrite in drinking water is less than 1 mg/L. Hence, the accurate monitoring of nitrite is also necessary. Several studies have researched using 2DMs for nitrite sensing. Saraf et al. reported nitrite sensors based on copper metal-organic frameworks and rGO (Cu-MOF/rGO) [98]. The Cu-MOF/rGO composite offers high conductivity to modify GC electrodes. A ST of 0.043736 AMc for the DR of 3–40,000 µM with a DL of 33 nm was reported. Jiao et al. demonstrated the operation of a nitrite sensor based on Au-RGO/poly(dially dimethyl ammonium chloride) composite [99]. The nanocomposite was produced by a facile one-pot method by utilizing PDDA as a reducing and stabilizing agent. The reported sensor has been used to test nitrite in water, meat, and dairy products. The sensor achieved a linear DR of 0.05–8.5 µM with a DL of 0.04 µM.

**Figure 9:**
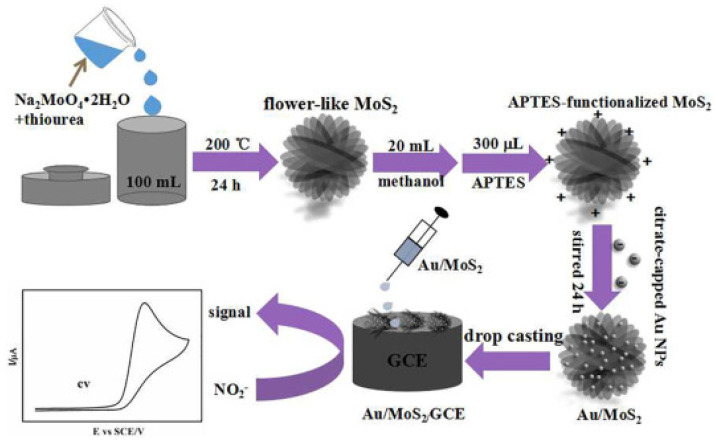
Synthesis of gold nanoparticles-MoS${}_{2}$ nanocomposite and gold nanoparticles-MoS${}_{2}$-GC electrode explained by the schematic. Reprinted with permission from Ref. [104].

Ma et al. developed a sensor by decorating ethanolamine and silver nanoparticles on GO for nitrite detection [100]. The ethanolamine was used to avoid agglomeration and achieve uniform silver nanoparticle distribution on GO. Using amperometry, a ST of 200 AMc for the DR of 0.05–3000 µM with a DL of 0.023 µM, was achieved. Other than metal particles, composites of organic polymers and graphene have also been used for nitrite sensors. Nie et al. prepared a nanocomposite of graphene and organic polymer Poly 3,4-ethylenedioxythiophene (PEDOT) using the electrodeposition method [101]. The nanocomposite has been prepared with a fairly simple fabrication method. A DL of 0.1 µM has been achieved for the linear DR of 0.3 to 600 µM. Another study based on graphene-PEDOT composite was carried out by Tian et al. Here, an electrode was made by using a 1D graphene-PEDOT nanocomposite coated with Tantalum film [102]. Graphene-PEDOT nanocomposites were prepared by electrochemical polymerization. 1D morphology resulted in a high catalytic activity with a DL of 7 µM for nitrite. Li et al. adopted a unique approach for nitrite detection using graphene quantum dots (GQD) [103]. A composite of GQD and N-doped carbon nanofibers (CN) was created by decorating GQD with CN using the electrospinning, annealing, and hydrothermal method. The GQD have a high density of defect sites but their conductivity has been improved by preparing the composite. This composite possesses advantages such as high defect density, large SA, and high conductivity. For this sensor, a linear DR of 5–300 µM and 400–3000 µM has been achieved with a DL of 3 µM. Other than graphene and its derivative, some other 2DMs were used for the detection of nitrite as well. In Ref. [104], Zhang et al. formed a composite of Au nanoparticles and MoS${}_{2}$ via the hydrothermal method, as demonstrated in Figure 9. The sensor possesses a linear DR of 0.005–27.8 mM, ST of 117.0 AMc with a DL of 1.67 µM.

Zhang et al. utilized the nanocomposite of Fe${}_{3}$O${}_{4}$ and MoS${}_{2}$ for the detection of nitrite [105]. A uniform distribution of Fe${}_{3}$O${}_{4}$ spheres was achieved on the MoS${}_{2}$ surface by the hydrothermal method on a GC electrode. The Fe${}_{3}$O${}_{4}$-MoS${}_{2}$ composite exhibit a higher electrocatalytic performance compared to only Fe${}_{3}$O${}_{4}$ or MoS${}_{2}$. With the amperometric response, a ST of 43.79 AMc, a DR of 1.0–2630 µM and a DL of 0.5 µM were obtained. Yang et al. covered MoS${}_{2}$ nanosheets with Nickel nanosheets to prepare a Ni-MoS${}_{2}$ composite for nitrite detection [106]. The composite was prepared and characterized using SEM, Raman spectroscopy, and XRD. A DR of 5–800 µM, and a DL of 2.48 µM with a ST of 214 AMc were achieved. Similarly, MXenes-based nitrite sensors are also studied. Zou et al. prepared a composite of 2D titanium carbide (Ti3C2TX) and Au NPs for nitrite detection [107]. The sensor exhibits good selectivity and ST for nitrite ions. A ST of 6.2 AMc with a DL of 0.14 µM and a low DL of 1.0 µM to 4581.1 µM has been achieved. Recently, Wang et al. Au prepared a composite of nanoparticles, Ti${}_{3}$C${}_{2}$Tx, and PDDA [108]. The composite benefits from the catalytic activity of Au NPs, the conductivity of Ti${}_{3}$C${}_{2}$Tx, and the electrostatic potential of PDDA. Using amperometry, a ST of 250 AMc with the linear DR of 0.1–2490 µM and 2490–13,490 µM and a DL of 0.059 µM was achieved.

## 4. 2DMs-Based Composite for Electrochemical Detection of Pesticides

Pesticides can contaminate human food and their long-term exposure is detrimental to human health. Studies have linked pesticides to Parkinson’s disease, and disorders in the reproductive as well as the endocrine system [109,110]. 2DMs possess tremendous potential to accurately detect harmful compounds such as organophosphorus (OPs) present in pesticides. The high electrical conductivity and the large SVR of 2DMs enhance the stability, detectivity, and catalytic activity of metal nanostructures. These qualities also help in the development of fixed suspensions by attempting to avoid agglomeration without the inclusion of surfactants or ligands, which is a need for the production of fixed suspensions. Using electrode approaches that were based on graphene, various compounds were found in the research field of food that dealt with OPs.

Setznagl et al. developed their sensor based on a GC electrode, modified with (GC/rGO-CuNPs) to detect glyphosate herbicide (GLY) using the DPV method. GLY was then used to estimate the amount of pesticide. The sensor exhibits a DR of 0.1 to 1.1 µmolŁ${}^{-1}$ with a DL of 0.19 µmolŁ${}^{-1}$ [111].

Yan et al. prepared a sensor based on hydroxyl (OH) groups of GO. With a response time of 1.8 s and a low DL of 0.21 µM, increased electrical as well as catalytic performance was attained. A DR of 0.43–218.40 µM was accomplished while maintaining a low DL [112]. Facure et al. studied the reduction of by the addition of conductive organic polymers (PEDOT:PSS and polypyrrole) and Au NPS. The sensor could successfully detect different OPs pesticides. A low DL of 0.19 µmolŁ${}^{-1}$ in 0.2 molŁ${}^{-1}$ PBS (pH = 7.0) was achieved [113]. The differential pulse voltammetric (DPV) approach with a ST of 33,270 AMc for carbaryl (CR) and 31,830 AMc for paraquat (PQ) was employed with a graphene altered boron-doped diamond electrode (BDDGR) published in Ref. [114] to effectively identify the carbaryl and paraquat pesticides.

In Ref. [115], carbofuran-phenol was measured by employing a screen-printed carbon electrode that was upgraded with Au NPs as well as GO (AuNPs/GO-SPCE). Additionally, the central composite design (CCD) technique was utilized to find the optimal conditions for conducting the experiment. Carbofuran-phenol was detected by DPV throughout a linear DR of 1–250 µM; the DL was approximately 0.22 µM.

**Figure 10:**
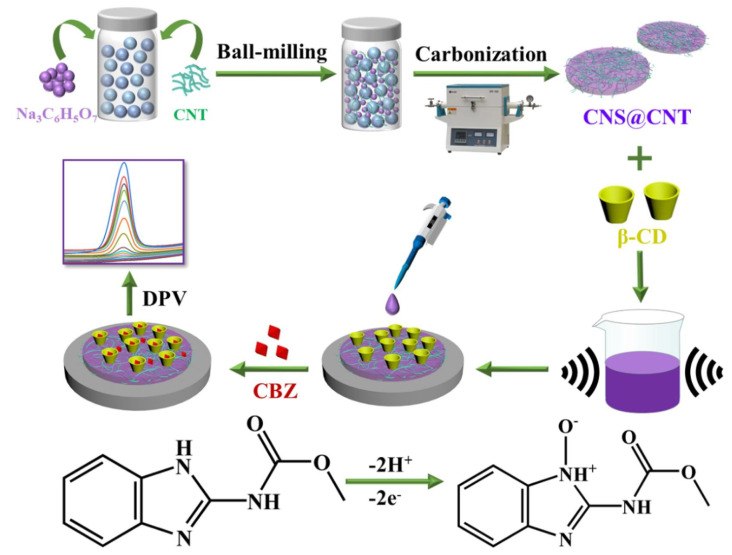
Schematic illustrating the fabrication steps of carbendazim sensor. Reprinted with permission from Ref. [118].

Xie et al. proposed an electrode material that is a composite of MXene (Ti${}_{3}$C${}_{2}$Tx) and electrochemically reduced graphite oxide (ERGO) for the electrochemical detection of carbendazim (CBZ) in orange juice and cucumber samples. The MXene/ERGO electrode was prepared by the green electrochemical approach. The sensor exhibits a good ST with a DR of 2 nM–10 µM and it shows a DL of 0.67 nM towards pesticide carbendazim detection [116]. Ozcan et al. detected carbendazim (CBZ) in water and food samples, CBZ is a common pesticide. The sensor was synthesized from a carbon paste electrode and a composite of Ag NPs on fumed silica. The sensor was tested on samples of apple, tomato, and orange juices along with the river water. The sensor can give stable results in a DR of $5.0\times 10^{-8}$ M–$3.0\times 10^{-6}$ M with a DL of $9.4\times 10^{-10}$ M [117]. Liu et al. reported a highly sensitive sensor for the detection of carbendazim (CBZ) in apple juice. The preparation scheme of the proposed sensor is shown in Figure 10. This sensor exhibits a linear DR of 0.03–30 µM with a high ST of 30,860 AMc. The reported DL is 9.4 nM [118].

Li et al. studied the quantification of the bactericide carbendazim (CBZ) in fruit and vegetable juice. The sensor is based on carbon nanohorns@rGO coated by gold platinum core-shell NPs. The performance evaluation was demonstrated on real samples of carrot and orange juice with satisfactory recovery ranges. A wide DR of 0.05–50 µmol/L with a DL of 1.64 nmol/L is achieved. The sensor’s ST is further improved due to the coating of gold platinum core-shell NPs [119].

**Figure 11:**
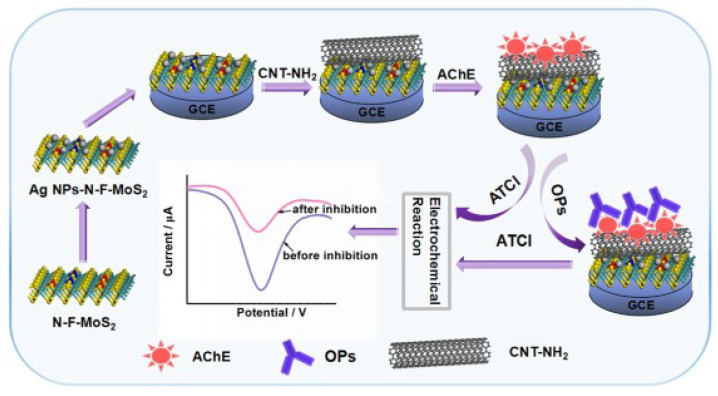
Schematic explaining the steps of fabrication of the pesticide sensor. Reprinted with permission from Ref. [120].

Ag NPs adorned with nitrogen and fluorine co-doped MoS${}_{2}$ (Ag-N-F-MoS${}_{2}$) alongside NH2-functionalized CNT-AchE enzyme-coated GCE were shown to be effective for monocrotophos and chlorpyrifos pesticides detection in a study conducted by Song et al. [120]. An oversimplified diagram of the process of creating an electrochemical biosensor for the detection of pesticides is shown in Figure 11. While pure MoS${}_{2}$ and AgMoS${}_{2}$ both showed strong electroactivity, the proposed composite material outperformed them in terms of electron mobility and specific SA. The biosensor has a LOD of 0.2 pM for monocrotophos and 3 pM for chlorpyrifos, and a DR of 0.4 pM–4 nM. Very little change is observed in the electrode response over a period of 30 days, which shows the stability of this proposed sensor. In addition to this, the authors hypothesized that the proposed electrochemical biosensor may be utilized for examination in real time.

Recent research reported the specific detection of methyl parathion (MP) based on MoS${}_{2}$ and graphene nanosheets. According to electrochemical impedance spectroscopy, the high sensing ability of MoS${}_{2}$-graphene/GCE was in agreement with the charge transfer resistance (EIS). As mentioned earlier, the charge resistance values of the MoS${}_{2}$-graphene-based electrodes were inferior to the bare/GCE electrodes, those modified with MoS${}_{2}$, and those changed with graphene. It is possible that this is due to the fact that when MoS${}_{2}$ is coupled with graphene nanosheets, the contact between the electrode and the electrolyte is significantly lessened [121].

A dual signaling mechanism, i.e., colorimetric and fluorometric, for the sensing of OPs pesticides, is proposed by Xie et al. in Ref. [122]. They have used gold nanoparticles (NPs) as the colorimetric probe and graphitic carbon nitride as the fluorescent probe to detect OPs. The sensor shows a good linear DR of $2\times 10^{-11}$ M to $6\times 10^{-9}$ M. It has a low DL as $6.9\times 10^{-12}$ M. The sensors is also tested to find the concentration of chlorpyrifos in fruit juices. Ouyang studied the g-C${}_{3}$N${}_{4}$@BiFeO${}_{3}$ nanocomposites as a single peroxidase-like catalyst to prepare the colorimetric/chemiluminescent dual-readout immunochromatographic assay to detect multiple pesticide residues [123]. This discussion about electrochemical sensors based on 2DMs for pesticides detection has been summarized in Table 2. For future sensors, the plasmons and quantum effects of 2D materials can be explored [124]. Further, the combination of 2D sensors with machine learning based on artificial neural networks can be a game changer for sensor networks [19]. Apart from these 2D materials, some other 2D materials such as black phosphorus also have vast potential for future electrochemical sensors and recent studies have investigated black phosphorus-based electrochemical sensors for applications such as the detection of antibiotics [125,126], glucose level sensing [127,128], cancer detection [129], and detection of food quality [130].

## 5. Conclusions and Future Perspectives

2DMs possess a strong potential to become the material of choice for future electrochemical sensors. 2DMs can create hybrid structures with other materials such as metal nanoparticles or bulk materials to enhance their sensing and chemical activity. Here, we have focused on the preparation and sensing mechanism of various 2DMs and their nanocomposites as well as the performance of sensors. On the other hand, there are certain areas that still need a lot of improvement. For example, it is challenging to grow a large area with uniform thickness and defect-free 2DMs. This makes it very difficult to obtain consistent reproducible results from the sensors based on 2DMs. Novel methods need to be developed to create better integration between existing materials and 2DMs. Finally, ultrathin 2DMs lose their intrinsic properties over time. It is also essential to develop techniques to store 2D materials for a longer period of time without the loss of intrinsic properties. Although 2DMs-based sensors have not been commercialized yet, from the amount of research that is going on in this area one can hope that the vast potential of 2DMs will soon be utilized for practical applications.

## Figures and Tables

**Table 1:** Comparison of different graphene-based electrodes for glucose detection.

Sensitivity (µA mM${}^{-1}$ cm${}^{-2}$)	Range (mM)	Detection Limit (µM)	References
85	0.005–1.270	1.73	[63]
48	0–2.40	5	[64]
26.50	0.50–13.50	1.30	[65]
5.40	0.10–12	17.80	[66]
1055	0–4	—–	[67]
—–	0.01–0.30	2.80	[68]
13,800	0.25–13.20	0.042	[69]
4.20	0.10–18	5.90	[70]
20.16	0.20–5.50	0.13	[71]
2300	5 nM–5 mM	0.0025	[72]
30	2–20	—–	[73]
171.92	0.02–5	6.30	[74]
2760	0.00001–0.050	790	[75]
256.60	0.005–8.20	2.70	[76]
11.06	0.01–3	2.83	[82]
64.75	0.002–4.096	0.53	[83]
312	0.002–28.33	0.42	[84]

**Table 2:** Numerous 2D materials based electrochemical sensors for the detection of pesticides.

Target Analyte	Range (µM)	Detection Limit (µM)	Matrix	References
Glyphosate	0.10–1.10 µmol/L	0.19 µmol/L	—	[111]
Dichlorvos	0.43–218.40	0.21	Green vegetable leaf	[112]
Carbaryl	1–6	0.07	Apple juice	[114]
Paraquat	0.20–1.20	0.01	Apple juice	[114]
Carbofuran	1–250	0.22	Cucumber, Rice	[115]
Carbendazim	0.002–10	$0.67\times 10^{-3}$	Cucumber, Orange Juice	[116]
Carbendazim	$5\times 10^{-2}$ – 3×10	$9.40\times 10^{-4}$	River water, Apple juice, Tomato juice, Orange juice	[117]
Carbendazim	0.03–30	$9.40\times 10^{-3}$	Apple juice	[118]
Carbendazim	0.05–50 µmol/L	1.64 nmol/L	Carrot juice, Orange juice	[119]
Chlorpyrifos	0.30–3 m	$3\times 10^{-6}$	—	[120]
Monocrotophos	$0.40\times 10^{-6}$–$4\times 10^{-3}$	$0.20\times 10^{-6}$	—	[120]
Methyl Parathion	$10\times 10^{-3}$–$1.90\times 10^{3}$	$3.20\times 10^{-3}$	Apple, Kiwi, Tomato, Cabbage	[121]
Chlorpyrifos	$2\times 10^{-5}$–$6\times 10^{-3}$	$6.90\times 10^{-6}$	Fruit juice	[122]
Methyl Parathion	$1\times 10^{-6}$–$1\times 10^{-2}$	$3.10\times 10^{-7}$	Apple Juice, Water	[131]
Carbaryl	0.10–12	0.02	Tomato, Grapes	[132]

## Data Availability

Not applicable.
